# Barriers to and opportunities for advancing racial equity in cervical cancer screening in the United States

**DOI:** 10.1186/s12905-024-03151-7

**Published:** 2024-06-21

**Authors:** Madina Agénor, Madeline Noh, Rose Eiduson, Merrily LeBlanc, Emmett C. Line, Roberta E. Goldman, Jennifer Potter, S. Bryn Austin

**Affiliations:** 1 Department of Behavioral and Social Sciences, Brown University School of Public Health, Providence, RI USA; 2 Center for Health Promotion and Health Equity, Brown University School of Public Health, Providence, RI USA; 3 Department of Epidemiology, Brown University School of Public Health, MPH Box G-S121-4, Providence, RI 02912 USA; 4 The Fenway Institute, Fenway Health, Boston, MA USA; 5 Larner College of Medicine, University of Vermont, Burlington, VT USA; 6 Department of Sociology, Northeastern University, Boston, MA USA; 7 Teachers College, Columbia University, New York, NY USA; 8 Department of Family Medicine, Warren Alpert Medical School, Brown University, Providence, RI USA; 9 Department of Medicine, Harvard Medical School, Boston, MA USA; 10 Department of Medicine, Beth Israel Deaconess Medical Center, Boston, MA USA; 11 Department of Social and Behavioral Sciences, Harvard T.H. Chan School of Public Health, Boston, MA USA; 12 Division of Adolescent and Young Adult Medicine, Boston Children’s Hospital, Boston, MA USA; 13 Department of Pediatrics, Harvard Medical School, Boston, MA USA

**Keywords:** Cervical cancer, Screening, Clinical guidelines, Racism, Health equity, Health care providers

## Abstract

**Background:**

In the United States (U.S.), racially minoritized people have higher rates of cervical cancer morbidity and mortality compared to white individuals as a result of racialized structural, social, economic, and health care inequities. However, cervical cancer screening guidelines are based on studies of predominately white individuals and do not substantially discuss or address racialized cervical cancer inequities and their social determinants, including racism.

**Methods:**

We conducted in-depth interviews with health care providers (*N* = 30) and key informants with expertise in health equity (*N* = 18). We utilized semi-structured interview guides that addressed providers’ views and experiences delivering cervical cancer screening to racially minoritized individuals and key informants’ recommendations for advancing racial equity in the development and implementation of cervical cancer screening guidelines. Interviews were analyzed using a template style thematic analysis approach involving deductive and inductive coding, memo writing, and matrix analysis for theme development.

**Results:**

Most health care providers adopted a universal, one-size-fits-all approach to cervical cancer screening with the stated goal of ensuring racial equality. Despite frequently acknowledging the existence of racialized cervical cancer inequities, few providers recognized the role of social inequities in influencing them, and none discussed the impact of racism. In contrast, key informants overwhelmingly recommended that providers adopt an approach to cervical cancer screening and follow-up care that recognizes the role of racism in shaping racialized cervical cancer and related social inequities, is developed in partnership with racially minoritized communities, and involves person-centered, structurally-competent, and trauma-informed practices that address racially minoritized peoples' unique lived experiences in historical and social context. This racism-conscious approach is not to be confused with race-based medicine, which is an essentialist and racist approach to health care that treats race as a biological variable rather than as a social and political construct.

**Conclusions:**

Developers and implementers of cervical cancer screening guidelines should explicitly recognize and address the impact of racism on cervical cancer screening, follow-up care, and outcomes, meaningfully incorporate racially minoritized communities' perspectives and experiences, and facilitate provider- and institutional-level practices that foster racial equity in cervical cancer.

## Background

Cervical cancer is one of the most common cancers around the world, with recent estimates approximating 570,000 cases globally [1, 2]. In the United States (U.S.), cervical cancer is far less common because of the widespread availability of Pap and human papillomavirus (HPV) testing in many areas [1–4]. Yet, the American Cancer Society estimates that 13,960 U.S. individuals will be diagnosed with cervical cancer, and 4,310 will die of the disease in 2023 [3]. Moreover, cervical cancer morbidity and mortality are unequally distributed among racialized groups in the U.S. From 2000 to 2018, cervical cancer incidence rates were highest among Black and Latina women at 16.8 and 15.8 per 100,000, respectively, compared to American Indian and Alaska Native women at 11.9, white women at 10.0, and Asian American and Pacific Islander women at 9.7 per 100,000 women [5]. Further, Black, American Indian and Alaska Native, and Latina women, among whom cervical cancer mortality rates were 5.0, 4.0, and 3.5 per 100,000, respectively, experienced higher mortality rates from the disease compared to white (2.6 per 100,000) and Asian American and Pacific Islander (2.1 per 100,000) women in 2000–2018 [5]. Additionally, Black women experience lower cervical cancer survival rates [1] and are more likely to be diagnosed with the disease at later stages [6, 7] relative to white women.

Racialized health inequities should be understood in the context of interconnected structural, institutional, cultural, and interpersonal racism, a historically-contingent social, economic, and political system that confers power to dominant racialized groups and undermines access to social, economic, and health care resources that are positively linked to the prevention and early detection of cervical cancer, including education, employment, income, health insurance, and regular access to care, among racially minoritized groups [1, 8–13]. These groups include Black, Native, and Asian populations, as well as Latine individuals who are racially minoritized based on their race, skin color, nativity, and/or social class [14–16]. In particular, research suggests that, as a result of racism-related barriers both inside (e.g., experiences of health care discrimination, concerns about vaccine safety, bias in provider HPV vaccine recommendation) [17–23] and outside (e.g., poverty, residential segregation) [18, 24] of the health care system, Black women in particular may be less likely than their white counterparts to complete the HPV vaccination series, which may in turn increase their susceptibility to cervical cancer.

Additionally, racism also increases Black and other racially minoritized women’s cumulative exposure to daily psychosocial stressors, including poor treatment in employment, educational, and health care settings, which may increase susceptibility to developing cervical cancer from high-risk HPV infection as a result of weathering processes, such as allostatic load and lowered immunity, that undermine HPV clearance [11, 25–27]. Further, reflecting racially segregated social contexts and sexual networks shaped by structural racism [28]—that is, the “the totality of ways in which societies foster racial discrimination through mutually reinforcing systems of housing, education, employment, earnings, benefits, credit, media, health care, and criminal justice” [29]—researchers have identified inequities in the prevalence of cancer-causing HPV types among racialized groups [30]. Moreover, data indicate that HPV types covered by the HPV vaccine may be less prevalent among Black women and, conversely, cancer-causing strains not covered by the vaccine may be more common among Black women relative to white women [30].

Access to and utilization of cervical cancer screening and timely follow-up care play an important role in shaping cervical cancer outcomes [4, 31, 32]. Cervical cancer screening tests include cervical cytology (i.e., Pap testing), primary human papillomavirus (HPV) testing, and Pap-HPV co-testing, all of which are associated with lower levels of cervical cancer morbidity and mortality [33]. Prior research shows that, on average, Black and white U.S. women have similar rates of regular Pap test use; in contrast, other racially minoritized groups—including American Indian and Alaska Native, Latine, and Asian subgroups—are less likely to be screened relative to their white counterparts [1, 34–36]. However, a recent study found that Black and Latina women were more likely to receive Pap testing alone relative to Pap-HPV co-testing [37]. Moreover, Black as well as Latina, American Indian and Alaska Native, and Asian women were less likely than white women to receive timely follow-up care and treatment upon an abnormal Pap test [38, 39]. Researchers have attributed such racialized inequities to social and health care factors linked to structural, institutional, and interpersonal racism, including a lack of access to preventive health services, limited financial resources to pay for prohibitive health care costs, and racial residential segregation [40, 41].

Health care providers are central to facilitating, or hindering, access to and utilization of cervical cancer screening and follow-up care. Indeed, studies show that provider efforts to recommend cervical cancer screening to patients through education, counseling, reminders, and invitation letters are associated with greater uptake [42–44]. In the U.S., providers typically follow clinical screening guidelines from institutions such as the American College of Obstetricians and Gynecologists (ACOG) [45, 46], ACS [47–49], American Society for Colposcopy and Cervical Pathology (ASCCP) [50, 51], and U.S. Preventive Services Task Force (USPSTF) [52]. All of these guidelines recommend that Pap tests be performed alone every three years among individuals with a cervix aged 21–29 years or every five years as part of Pap-HPV co-testing among individuals aged 30–65 years [53]. In recent years, the Black Women’s Health Imperative (BWHI), the only national organization solely focused on advancing health equity for Black women, has raised concerns that screening guidelines generally do not reflect the specific lived experiences and historical and social contexts of Black women [54]. In particularly, they warned that screening guidelines advance a one-size-fits-all approach that may not be appropriate for racially minoritized individuals at greater socially constructed risk of cervical cancer [41, 54–59].

Indeed, despite pronounced racialized cervical cancer inequities, existing screening guidelines are largely based on studies conducted among white individuals [60, 61] and minimally discuss the disproportionate burden of cervical cancer morbidity and mortality among disaggregated racially minoritized groups, the role of racism in shaping racialized cervical cancer inequities, or strategies to address them [45–52, 62]. For example, the ACOG guidelines note that communities of color and other underserved groups may have limited access to primary HPV testing and thus encourage retaining cytology-based screening in screening recommendations until HPV testing is more widely accessible [46]. The ACOG guidelines also recommend HPV self-sampling as a strategy to increase access to screening among these populations. However, the guidelines do not disaggregate racially minoritized groups with unique and specific experiences or address the role of social determinants of health, including racism, in shaping racialized inequities in cervical cancer screening follow-up care, or outcomes [46].

Moreover, although the American Cancer Society recognizes the disproportionate burden of cervical cancer among racially minoritized groups, their guidelines focus on addressing individual-level behavioral factors (namely, differential health-seeking behaviors) rather than the social and economic drivers of racialized cervical cancer inequities in the context of structural racism [47–49]. Additionally, the ASCCP guidelines recommend that racially minoritized patients be screened with Pap/HPV co-testing until primary HPV testing is more widely accessible [50]. Further, they note that primary HPV testing may not account for the HPV subtypes frequently detected among Black and Latine individuals. Although the ASCCP guidelines mention factors contributing to higher rates of cervical cancer among Black populations (e.g., lower follow-up rates upon abnormal screening results), they are not explicitly tied to social inequities in the context of racism [50]. Lastly, while the USPSTF guidelines describe racialized inequities in cervical cancer outcomes, they include limited discussion on the role of racism in shaping them or on recommendations to address them [52].

Given health care providers’ central role in cervical cancer screening, it is imperative to understand how providers implement screening guidelines among racially minoritized people. Prior research has examined providers’ perspectives and attitudes toward considering race, racism, and health equity in their clinical decision-making [63–65]. Some of these studies show that many providers are subject to bias against Black, Latine, and other racially minoritized people, which negatively influences judgement in clinical encounters [66, 67]. In turn, these biases produce harmful effects, such as eroding the quality of patient-provider communication, lowering treatment follow-up among patients, and increasing provider usage of negative descriptors for patients of color in electronic health records, which further perpetuates discrimination [67–69]. Additionally, other studies indicate that many providers fail to address the specific social circumstances of racially minoritized patients in the context of racism and other forms of discrimination, leading to a lack of appropriate cervical cancer screening counseling and care [70–73].

However, to our knowledge, no prior study has specifically examined health care providers’ perceptions, attitudes, and experiences related to addressing racialized cervical cancer inequities and promoting health equity—which requires valuing all individuals and populations equally, recognizing and rectifying historical injustices, and providing resources according to need [74]—in the context of cervical cancer screening. Thus, we conducted a qualitative research study to: (1) better understand how providers make decisions about cervical cancer screening counseling and care among patients from racially minoritized backgrounds and (2) identify the beliefs, attitudes, and experiences that drive their clinical decision-making. Moreover, we sought to address the notable lack of research-based recommendations for advancing racial equity in cervical cancer screening by seeking the input of leading national experts in this understudied area.

## Methods

### Study population

We conducted in-depth online interviews with 30 health care providers who were recruited from Massachusetts and Rhode Island, employing a purposive sampling approach to identify eligible participants based on the following criteria: works as a physician, physician assistant, or nurse practitioner; practices in a non-emergency setting at a health care facility; provides care to people aged 18 years and above in Massachusetts or Rhode Island; and has administered an HPV vaccine and/or Pap test in the past year. We selected Massachusetts and Rhode Island as our recruitment sites because doing so allowed us to efficiently recruit 30 health care providers for our study using our professional networks and provided an opportunity to more directly influence policy recommendations. Additionally, we recruited 18 public health and health care professionals (not necessarily practicing) with expertise in health equity for racialized, sexual, and gender minoritized groups to participate in key-informant interviews. We used a purposive sampling approach to select individuals from across the U.S. with experience providing leadership on issues of health equity related to race/ethnicity, sexual orientation, and gender identity in population health or research, practice, or funding. Notably, we conducted key-informant interviews to further explore the topic of clinical guidelines addressed in the provider interviews and elicit expert recommendations on how to incorporate equity into the development and implementation of cervical cancer screening guidelines. We utilized our professional networks to identify and recruit (by email) health care providers and key informants at community health centers and hospitals in Massachusetts and Rhode Island as well as academic institutions, policy and community-based organizations, government agencies, and foundations. We also relied on snowball sampling, wherein study participants shared information about the study with their colleagues, and used maximum variation sampling based on topic and place of practice to obtain a sample of providers and key informants from diverse professional backgrounds [75–78]. We stopped recruitment of both providers and key informants when we achieved thematic saturation with regard to the study’s research questions pertaining to provider perspectives and experiences related to cervical cancer screening and prevention among AFAB patients with minoritized racialized, sexual orientation, and gender identities as well as key informants’ recommendations on advancing health equity in the context of cervical cancer screening guidelines [79].

### Data collection

Health care provider interviews were conducted in June and July 2020, and key informant interviews with health equity experts took place from March to May 2021. All interviews were conducted using Zoom and lasted approximately 60 min. Prior to beginning the interview, each participant filled out a brief survey with questions on their demographic characteristics and provided informed consent verbally. Health care provider and key-informant interviews were conducted using two separate semi-structured interview guides containing open-ended questions, which were formulated based on the existing scientific literature on bias and equity in health care providers’ recommendation and delivery of clinical services and provider- and institutional-level determinants of cervical cancer screening among patients with minoritized racialized sexual orientation, and gender identities. Additionally, our interview guides were informed by the World Health Organization conceptual framework for action on the structural and social determinants of health [80], which specifies that health care factors are shaped by both structural and social determinants of health, including racism. Both guides were revised based on feedback from experts in cervical cancer, health equity, clinical counseling and services delivery, racial bias in health care, sexual and gender minority (SGM) health, and qualitative research methods. The provider guide addressed the following broad topical areas: previous training in racial/ethnic, sexual orientation, and gender identity equity in health care, approaches to sexual history taking, patient-provider communication in the context of cervical cancer screening, and cervical cancer prevention counseling and services delivery among patients with minoritized racialized, sexual orientation, and gender identities. The key-informant guide discussed the content, development, and implementation of screening guidelines in the context of racial/ethnic, sexual orientation, and gender identity equity as well as recommendations for advancing health equity. Participants were given a $100 gift card for their time. All research activities were approved by the Tufts University Social, Behavioral, and Educational Research Institutional Review Board.

### Data analysis

A professional transcription company created verbatim transcripts of all interview recordings. These transcripts were then uploaded to Dedoose (Version 9.2.007, Manhattan Beach, CA) to enable team-based qualitative data analysis. We used a template style thematic analysis approach rooted in a critical realism research paradigm, which specifies that different people view social phenomena caused by multiple, context-specific, (un)observed mechanisms from different subjective perspectives [81], to develop themes and sub-themes that offer potential explanations regarding observed patterns of phenomena of interest [75, 82–84]. We first developed two initial hierarchical codebooks, one for the provider interviews and another for the health equity key informant interviews. These codebooks were created based on inductive codes identified using interview transcripts as well as deductive codes conceptualized from the scientific literature and interview guides [82–87]. The research question guiding the development of the provider codebook was: What are health care providers’ views, experiences, and behaviors delivering cervical cancer screening to racially minoritized patients? The research question guiding the key informant codebook was: What recommendations do health equity experts have for incorporating racial equity into the development and implementation of cervical cancer screening guidelines?

Two sets of independent coders then iteratively refined each codebook by applying each of the two initial codebooks to a subset of health care provider and key-informant interview transcripts to test their fit to the data [82–87]. The coders then independently applied each codebook to the relevant set of interviews, double coding a subset (20%) of transcripts to ensure consistent application of the codebooks among coders and revising the codebook as needed [82–87]. The coders for each set of interviews met regularly to discuss preliminary themes, compare coding patterns, and resolve coding discrepancies through consensus [82–87]. The present analysis pertains solely to codes related to barriers to and opportunities for advancing racial equity in cervical cancer screening; findings related to sexual orientation- and gender identity-related equity are presented elsewhere. Code summaries were produced for relevant coded excerpts, which were clustered into separate themes and sub-themes for providers and key informants using each hierarchical codebook, memo writing, and team-based discussions [83, 84, 86, 88]. Additionally, we employed data analysis matrices to facilitate subgroup comparisons, including in relation to professional background and race/ethnicity, among providers [89].

## Results

Participant demographic characteristics for health care providers and key informants are presented in Tables 1 and 2, respectively. Below, we present three themes pertaining to racial equity in cervical cancer screening; the first two were developed using health care provider interviews whereas the third was developed using key informant data. Because key informant interviews were specifically conducted to elicit expert recommendations on a topic identified in the health care provider interviews (i.e., how to incorporate equity into cervical cancer screening guidelines), themes derived from provider and key informant data are presented in tandem (as they are complementary) but are not integrated (as they are derived from distinct datasets).

**Table 1:** Sociodemographic and professional characteristics of health care providers (*N* = 30)

Characteristic	*n*	%
Age (years)		
18–30	6	20
31–40	16	53
41–50	4	13
51–63	4	13
Sex assigned at birth		
Female	28	93
Male	2	7
Gender identity		
Man	2	7
Woman	28	93
Race/ethnicity^a^		
Asian/Asian American or Pacific Islander	6	20
Black	4	13
Latine	2	7
Multiracial	1	3
White	20	67
Sexual orientation identity^a^		
Bisexual	3	10
Gay or lesbian	3	10
Heterosexual	24	80
Queer	4	13
State of practice		
Massachusetts	25	83
Rhode Island	5	17
Health care setting		
Clinic	11	37
Community health center	8	27
Hospital	10	33
Private practice	1	3
Geographic location		
Suburban	3	10
Urban	27	90
Profession		
Nurse practitioner	8	27
Physician (MD or DO)	14	47
Physician assistant	8	27
Specialty		
Adolescent/young adult medicine	4	13
Family medicine	12	40
Internal medicine/primary care	5	17
Obstetrics/gynecology/reproductive health/women’s health	12	40
Pediatrics	3	10
Length of practice experience (years)		
<1	1	3
1–5	16	53
6–10	7	23
>10	6	20

*Note.* Percentages may not add to 100% due to rounding error

^a^Categories are not mutually exclusive

**Table 2:** Sociodemographic and professional characteristics of health equity key informants (*N* = 18)

Characteristic	*n*	%
Sex assigned at birth (*n* = 17)		
Female	14	82
Male	3	18
Gender identity (*n* = 17)		
Man	2	11
Nonbinary	1	6
Woman	14	78
Race/ethnicity^a^		
Asian/Asian American or Pacific Islander	2	12
Black	3	18
Native	1	6
White	10	59
Another race/ethnicity	1	6
Sexual orientation identity^a^		
Gay or lesbian	3	17
Heterosexual	12	67
Queer	4	22
Highest degree earned		
Doctoral (PhD, ScD, DrPH)	7	39
Master’s (MA, MS, MPH, MPP)	9	30
Medical (MD, DO)	9	50
Geographic region		
Midwest	1	6
Northeast	7	41
South	6	35
West	3	18
Place of work		
Academic institution	9	50
Health care facility	7	39
Research organization	4	22
Other (policy, philanthropy, health department)	2	11
Health equity focus^a^		
LGBTQ+	9	50%
Racial/ethnic	9	50%
Other (socioeconomic, global)	3	18%

*Note*. Percentages may not add to 100% due to rounding error

^a^Categories are not mutually exclusive

### THEME 1. Adopting a one-size-fits-all approach to cervical cancer screening

#### Sub-theme 1. Adhering to universal clinical guidelines

The vast majority of health care providers reported adopting a universal approach to cervical cancer screening. Indeed, almost all providers reported strictly adhering to established guidelines in their Pap test recommendation and delivery practices with all patients. For example, echoing most providers’ views, a white female nurse practitioner noted: “I try not to allow demographic characteristics to impact those decisions because that, according to ACOG [American College of Obstetricians and Gynecologists], should not come into play. It is just the recommendation.” Several providers affirmed that, although they account for age and Pap test history, as indicated by screening guidelines, they do not consider other social factors—such as race/ethnicity, which they conceptualized as an individual-level demographic characteristic—in their screening decisions. For example, a white female physician observed: “There are no race-based recommendations that I’m aware of in terms of […] cervical cancer screening. And so that doesn’t make a difference for me.” Moreover, a Latina female nurse practitioner explained: “They don’t give me different algorithms for race. […] They give me algorithms based on last Pap [test] and age. So, that’s what I follow.”

#### Sub-theme 2. Refraining from applying population health inequities data to individual patient care

Most health care providers were aware of research identifying pronounced racialized inequities in cervical cancer outcomes. However, they stressed that population-level health inequities data should not inform individual patient care. Some providers justified this argument by underscoring the importance of following screening guidelines rather than relying on population health data. For example, a white female nurse practitioner stated: “I think I am sort of hyperaware of the fact that there are a lot of health disparities.” She continued, “I do not think that race or ethnicity should come into play in those [cervical cancer screening] decisions. I think the recommendations are the recommendations.”

Additionally, a few providers emphasized the importance of engaging in person-centered communication about screening with all patients, including those from racially minoritized backgrounds, instead of applying group-level data to individual-level clinical decisions. For example, an Asian female physician noted: “I think with an individual patient encounter, it doesn’t matter so much. […] I think this data that’s by population level can be useful but can also maybe not apply in the same way when you’re having individual conversations.” Further, a few providers emphasized not relying on population health inequities data in the context of clinical care so as to provide each patient, regardless of race/ethnicity, with the most effective possible intervention against cervical cancer. For example, a Black female physician assistant noted: “I think in general, in the literature, the science tends to point us to women of African descent and women of Latino background being more at risk than Caucasian women. […] But when I’m thinking about women’s health, especially Pap smears, I am looking at this woman as: ‘This is what has been shown and has been helpful for cervical cancer. This is what I’m offering you.”

#### Sub-theme 3. Prioritizing racial equality in cervical cancer screening

Some providers invoked the principles of racial equality in their rationale for adopting a one-size-fits-all approach to cervical cancer screening based on universal clinical guidelines. For example, a Black female nurse practitioner explained: “I try to treat everybody the same regardless of their race. They should all…we should be following the guidelines the same for everybody across the board.” She continued: “You shouldn’t look at who’s in front of us just trying to decide oh, because this or that, they need a Pap, or they don’t need a Pap. You treat them all kind of equally.” Notably, a few providers expressed concern that taking into account information on racialized inequities in cervical cancer outcomes when making cervical cancer screening decisions could result in unequal care (namely, underscreening) for white women. For example, a white female physician explained: “My understanding is that maybe rates of cervical cancer are a little higher in non-white women. But, in any case, I wasn’t going to not recommend Pap screening for white women.”

### THEME 2. Conceptualizing and considering race/ethnicity and racism in cervical cancer screening

#### Sub-theme 1. Race/ethnicity as an indicator of socioeconomic position and access to care

Although the vast majority of providers conceptualized race/ethnicity as an individual-level social demographic characteristic—and most reported not considering patients’ race/ethnicity or racialized health inequities data in their screening decisions—some providers discussed race/ethnicity as a social factor that can shape cervical cancer screening. For example, an Asian female physician noted: “I think the data shows that Black and Hispanic women are more likely to have cervical cancer. But I think that’s more a question of access and not necessarily related to individual behavior.” Similarly, another Asian female physician observed: “I wonder, in terms of accessing care regularly or things like that, whether people base it on SES [socioeconomic status], which seems to be so tightly linked to race.” She continued: “If you are not coming in for routine exams, then I feel like that [cervical cancer screening] is going to totally get missed.”

Relatedly, in describing how they take race/ethnicity into account in their screening practices, a few providers mentioned tailoring their approach to address patients’ access to socioeconomic resources, health insurance, and regular source of care, which they recognized as being correlated to race/ethnicity. For example, referring to uninsured patients, whom she described as being disproportionately Latine immigrants, a white female physician assistant explained: “Sometimes, I future order stuff. I’ll order testing that they can get anytime in the next three months and tell them, ‘Look, whenever you’re able to pay for it, or you have insurance, or you’d like to get it done, just come back, and we can do it.”

#### Sub-theme 2. Racism: the missing link

Although these providers acknowledged the on average relationship between race/ethnicity and access to socioeconomic and health care resources, which is driven by structural, institutional, and interpersonal racism [13], they did not explicitly mention racism. However, one provider discussed acknowledging the effects of racism on Black and other patients of color’s discomfort with and lack of uptake of Pap tests. Specifically, a white female nurse practitioner reported addressing the historical legacy of racism in gynecology in her conversations about Pap tests with Black and other patients of color: “I am much more deliberate about explaining what a Pap smear is for any person of color because there has been a very dark history of coercion when it comes to gynecologic health for people who are of color and Black people.” She continued: “Instead of saying you need a Pap and that is it, I’m going to spend a lot more time explaining to a person that it is a pelvic exam, it is a screening. I will tell them exactly how it is done, what it is for, where the cells are going. I tend to spend a lot more time talking to women of color about the Pap than I do white women.” Although this approach importantly addresses the ongoing psychosocial effects of historical racism on Black people and other people of color’s cervical cancer screening experiences, it does not address the impact of historical and contemporary racism on patients’ ability to access high-quality, person-centered screening and follow-up care that affirm their humanity, bodily autonomy, and dignity.

### THEME 3. Recommendations for advancing racial equity in cervical cancer screening

#### Sub-theme 1. Centering racial equity in cervical cancer screening guideline development

Most key informants in our study emphasized that health equity should be centered at all stages of the screening guidelines development process. In particular, key informants recommended that the racialized communities most impacted by cervical cancer, including Black, Latine, Native, and other people of color, be actively engaged in guideline development, including by being invited to join expert panels and community advisory boards. Stated goals of community engagement in guideline development included ensuring that screening guidelines reflect the needs, concerns, priorities, and experiences of racially minoritized groups and contribute to building trust between health care institutions and minoritized communities. For example, a key informant recommended: “I think it’s critical to have representation, just like I think it’s critical to have representation [and] inclusivity on the panel. Like making sure the expert panel is diverse in discipline, lived experience, research expertise.” Similarly, another key informant noted: “Yes, we need to adjust all of those boards so that we are more inclusive of the people that [experience cervical cancer inequities]. We call them advisory boards, but we’re not really being advised by people who understand differences [in cervical cancer screening experiences]. [We’re being] advised by, you know, if I’m being honest, in the past, it’s been largely white males, so we really need to look at a shift in this.”

Additionally, many key informants stressed that screening guidelines should be based not only on data from white individuals, as is often the case, but also on research that reflects the specific lived experiences of racially minoritized communities in social and historical context. For example, a key informant recommended “looking at cervical cancer guidelines and making them more responsive, at the very front end.” She explained: “Do we have enough data on populations who are at disproportionate risk for cervical cancer that we can make appropriate recommendations regarding the guidelines? Do we have enough data from racial/ethnic minority groups? […] Do we have enough data on social determinants of health [and] how those could impact cervical cancer risk and thereby screening? Then, taking that to the guidelines to inform how they’re tailored and potentially rewritten.” Of note, several key informants underscored the importance of basing guidelines on data from underrepresented groups (e.g., Asian), more broadly defined, as well as from specific, disaggregated ethnic subgroups (e.g., Chinese, Vietnamese, Korean) in order to account for the notable within-group heterogeneity in cervical cancer outcomes. For example, a key informant explained: “From an Asian American perspective, Asian and Pacific islander perspective, two thirds of our community are foreign born, and […] there is something like 50 different ethnicities. You can’t say that there’s one monolithic Asian American/Pacific Islander approach to anything. It really has to be for each ethnicity because each is a different culture. And the demographics and the stats are different; so, straight off the bat, the guidelines have to be adjusted for each.”

Further, many key informants recommended that screening guidelines be revised to address social determinants of health, including racism. For example, one key informant noted: “I would say, at the very least, maybe guidelines could have a general statement about treating people holistically and taking into consideration these other social determinants of health that might impact how or when somebody decides to receive care.” Several key informants also explicitly stated that cervical cancer screening guidelines should address the impact of racism on access to and utilization of screening. One key informant observed: “I think [that], at this stage in America, we’re really reconciling what racism has done, [including in] our healthcare system. I think it’s a moment in time—or this is when we should be sort of reframing how things are produced, and so no longer just kind of doing it [i.e., providing care] without taking these things into consideration. Because if it’s perpetuating it, it has to be seen as harmful. It can’t just be seen as benign or innocent to ignore racism or discrimination. It is not benign. It is in fact discriminatory and racist. And, in my opinion, to erase the impact of those things is another form of perpetuating them.” Recognizing barriers to addressing social determinants of health equity in clinical guidelines, another key informant recommended: “It may be challenging to incorporate social determinants and health equity specifically into a guideline statement. […] But even if it can’t change quickly, or they want to keep the way the system works, [it] doesn’t mean you can’t create a new guideline and both are required.”

#### Sub-theme 2. Ensuring racial equity in the implementation of cervical cancer screening guidelines

##### Tailoring the implementation of cervical cancer screening guidelines to promote racial equity

Most key informants noted that, because guidelines tend to rely on research based on samples of predominately white, heterosexual, cisgender women with high levels of education, a decontextualized, approach to cervical cancer screening and follow-up care may not be suitable for all patients. As such, many key informants recommended that, to adequately address the needs of racially minoritized patients, providers and institutions should tailor their approach to implementing cervical cancer screening guidelines by incorporating person-centered and structurally-competent strategies that help buffer the effects of structural, institutional, and interpersonal racism.

##### Provider-level strategies for promoting racial equity in cervical cancer screening

Most key informants recommended that health care providers utilize shared decision-making with racially minoritized patients, with the goal of ensuring bodily autonomy and agency in the context of racism. Recommended shared decision-making strategies included ensuring person-centered communication during screening, providing patients with additional information and counseling before or during exams, and establishing a collaborative, long-term screening plan that addresses patients’ concerns, preferences, lived experiences, and barriers to care. For example, one key informant mentioned: “[Patients] get to make the decision about where things go after that [i.e., once they’ve received relevant information]. So really sort of educating [them] in a way that gives them voice and autonomy over their health.”

Moreover, several key informants noted that health care providers should practice—and should be trained to use—trauma-informed approaches to screening with their racially minoritized and other socially marginalized patients to address the potential effects of racism and other forms of discrimination on patient trust and comfort. For example, a key informant explained: “If you’re from a Black or brown community, most likely you’ve experienced either some kind of social stigma, racism, discrimination—so there’s trauma there. If you’ve faced adverse childhood experiences, that’s trauma and then you add cancer, which is definitively a traumatic experience.” As a result, she recommended “having clinicians come to an encounter with the assumption, or not even the assumption, the openness that people have experienced multiple levels of trauma and how a particular procedure might trigger or exacerbate that trauma.” She continued: “Being sensitive to that, I think, is critical.”

Further, other key informants discussed the importance of providers challenging their biases towards racially minoritized patients and the causes of racialized health inequities. For example, with regard to providing care to patients with racially minoritized identities, one key informant recommended: “I think the biggest thing is [to] reflect on your immediate reactions and consider whether those reactions will be helpful to the patient. Recognize [your] biases and find ways to be open and work with the patients.” Moreover, pertaining to the root causes of racialized inequities in cervical cancer outcomes, screening, and follow-up care, several key informants noted that providers tended to attribute these inequities to race, which they conceptualized as a biological or behavioral phenomenon, rather than to racism—which shapes racially minoritized people’s risk of developing cervical cancer by increasing exposure to chronic stressors that increase weathering and decreasing access to social, economic, and health care resources. For example, a key informant observed: “Instead of saying racism, we say race. We can wrap [our] heads around that. […] It seems easier, we can put our finger on it. But what strikes me is that that’s the sort of medical way. We treat the symptom. But we are public health professionals. We ought to treat the true underlying [cause] from a preventative standpoint.”

##### Institutional-level strategies for promoting racial equity in cervical cancer screening

Key informants also recommended several institutional-level strategies for promoting racial as well as socioeconomic equity in screening, including expanding clinic hours. For example, a key informant mentioned: “There are different ways to make things easier. I work at a clinic [where] we offer evening hours and Saturday hours for folks [for whom] it’s difficult to come in for appointments, at least [during] traditional clinic hours. [It’s important] that they have different options and weekend options, to at least increase appointment availability…Expanding clinic hours is important.” With regard to advancing health equity in cervical cancer screening in particular, several key informants recommended that health care institutions employ strategies that promote patients’ bodily autonomy and agency, including self-administered HPV testing in the clinic or at home. Moreover, many key informants recommended that, to facilitate access to screening as well as follow-up care upon a positive diagnosis, health care institutions should address socioeconomic barriers to care, including cost, transportation, and health insurance.

Moreover, a few key informants noted that health care institutions should allow providers to alter guideline-determined screening intervals, including delaying screening until trust has been established between patient and provider. For example, a key informant noted: “If someone needs a Pap smear for cervical cancer screening because of their age, sexual history, and risk profile, and they don’t want to do it the day that I bring it up, we kind of make a plan to do it. I think, making sure you give them time to process what’s going to happen, explaining in detail how it’s going to be, and making sure you’re at a point in your relationship as doctor and patient where the patient feels comfortable with you performing that procedure. Usually, we get to a point where we can do it. [It] just may be delayed, and for screening, I don’t think that’s a big deal.” Conversely, several key informants of color noted that institutions should allow providers to shorten screening intervals to account for many patients of color’s current or anticipated limited access to care and insurance coverage. However, they warned that providers may face barriers to adopting this approach, notably from health insurance companies. For example, one key informant noted: “I don’t just want to say, ‘Oh no, ma’am, you’re actually not due until October. We’ll reschedule you, and you can do all that again.’ That’s just wrong. I mean, you know there’s no reason. But then, if I choose, as the health equity advocate, to go ahead and grab that Pap, she’s going to get a bill for it, and now [we’ve] harmed her again, you know.” She continued to explain: Maybe […] she can’t afford a bill for an uncovered procedure. So you’re kind of really jammed in there either way once it gets to payment. If it just stays in sort of the academic concept, here’s a guideline, take it or leave it…I think you can still make those judgments as a clinician, but unfortunately kind of run up against the risk of things not being paid for too.”

Other key informants noted that new health care facilities that center the needs of racially minoritized patients and are staffed by providers from the same racialized backgrounds are critical for promoting equity. For example, a key informant explained: “There are specialty Muslim clinics in LA. There’s the United Muslim Medical Association clinics. They do amazing work. And because all the practitioners are Muslim and all the staff are, they have figured out how to respect the cultural practices and still provide the standard of care.” Underscoring the importance of health care institutions hiring, supporting, and retaining health care providers of color, a few key informants noted that ensuring racial/ethnic concordance between patients and providers could positively impact the implementation of other provider-level strategies for advancing health equity, including shared decision-making. For example, one key informant observed: “Based on the length of communication and who talks and how much there is in terms of shared decision-making, there are very different patterns if it’s a white or Black provider.”

## Discussion

Most health care providers in our study adopted a universal, one-size-fits-all approach to cervical cancer screening based on guidelines developed using data from studies of predominately white individuals and without substantive attention to or input from racially minoritized communities [60, 61]. Indeed, although most providers acknowledged the existence of racialized cervical cancer inequities, few recognized the role of social inequities in shaping them, and none discussed the impact of racism. Notably, some providers argued that adopting a universal clinical approach that does not consider racism and related social inequities was a means of ensuring racial equality, emphasizing that all individuals should be treated the same. Nonetheless, a few providers recognized and considered the influence of social, economic, and health care inequities shaped by structural racism on cervical cancer screening, follow-up care, and outcomes among racially minoritized patients. However, racism itself was never explicitly mentioned.

In contrast, the key informants in our study recommended that cervical cancer screening guidelines themselves, as well as the processes through which they are developed and implemented, be changed to incorporate the experiences, contexts, and recommendations of racially minoritized and other socially and economically marginalized groups. In line with recent calls by USPSTF and others to address racialized health inequities and the role of racism through clinical guidelines [60–62, 90], key informants recommended that cervical cancer screening guidelines be formulated based on racially/ethnically disaggregated data that meaningfully consider the specific historical, structural, social, and economic contexts of diverse racially minoritized groups and subgroups, including the impact of racism on health and health care [60–62, 91–94]. Further, they suggested that the specific and unique lived experiences and social contexts of Black, Latine, Native, and Asian communities be centered when developing clinical guidelines in order to facilitate the delivery of equitable cervical cancer screening and follow-up care among diverse racially minoritized groups [55, 95, 96]. Finally, they noted that the process of formulating cervical cancer screening guidelines should be rigorously evaluated through a health equity lens and in partnership with racially minoritized communities [96].

Additionally, our study contributes to the existing literature on incorporating equity and addressing structural and social determinants of health, including racism, in the context of clinical guidelines by underscoring the importance of advancing these principles and practices in not only the development but also the implementation of guidelines. In particular, key informants recommended that clinical guidelines encourage providers to adopt a person-centered, structurally-competent, and trauma-informed approach to care that incorporates aspects of patients’ social history beyond age and Pap test history, including but not limited to socioeconomic position, health insurance status, access to care, educational level, language, social stressors, and environmental exposures [92, 97, 98]. Moreover, key informants made several institutional-level recommendations related to the implementation of screening guidelines to monitor and address barriers that disproportionately impact racially minoritized people, including altering screening intervals, providing transportation, and expanding clinic hours; reforming counseling protocols to incorporate person-centered communication and shared decision-making [99, 100]; and facilitating access to HPV self-sampling, both in the clinic and at home, which has been shown to be effective and supports patients’ bodily autonomy [101].

Our findings should be interpreted in the context of several limitations. First, although key informants were recruited nationally, health care providers were recruited from Massachusetts and Rhode Island only. Thus, our results based on providers’ accounts may not be applicable to those practicing in states with different social, economic, and political contexts. In particular, it is possible that providers working in areas with higher burdens of cervical cancer (e.g., Puerto Rico, Southeastern U.S.) may have different approaches to cervical cancer screening than those in areas with lower rates. Yet, the implications of our findings related to addressing racism in the context of cervical cancer screening are applicable to all areas of the U.S. given similar histories and practices of settler colonialism, white supremacy, and marginalization towards racially minoritized people across geographic regions [102–106].

Second, the providers in our study were largely white, heterosexual, cisgender women, which reflects the distribution of providers in the U.S [107–110] but limits the transference of our findings to providers with other social identities. Nonetheless, one-third and nearly one quarter of providers were from racially and sexual minoritized backgrounds, respectively. Additional research is needed to better understand the experiences of providers with social identities that were underrepresented in our sample, including providers of color and lesbian, gay, bisexual, transgender, queer, and other sexual and gender minoritized (LGBTQ+) providers. Third, most of the providers we interviewed were 31–40 years of age and had one to five years of clinical practice experience. Thus, our findings may not reflect perspectives of providers with additional years of experience. Fourth, although our study drew on data from both health care providers and key informants, we utilized a singular approach to data collection—namely, in-depth interviews—and did not integrate our findings since key informant interviews were specifically conducted to elicit expert recommendations on a topic identified in the health care provider interviews (i.e., how to incorporate equity into cervical cancer screening guidelines) and not to be triangulated with provider data. To facilitate data triangulation, future research should employ multi-method qualitative study designs as well as mixed-methods research approaches. Lastly, the present manuscript focused on health equity for racially minoritized people in particular, and our findings on addressing sexual orientation- and gender identity-related inequities in the context of cervical cancer screening have been reported elsewhere. Future research should adopt an intersectional approach to elucidate how racism and other forms of discrimination related to socioeconomic position, gender identity, sexual orientation, immigrant status, and weight, among others, simultaneously influence screening in mutually influencing and compounding ways.

Findings suggest that the development and implementation of cervical cancer screening guidelines should explicitly recognize and address the impact of racism, actively involve racially minoritized communities, and facilitate provider- and institutional-level practices that foster racial equity in cervical cancer outcomes. Moreover, in their screening efforts, health care providers should acknowledge racialized cervical cancer inequities as well as the role of racism in shaping them [56, 70, 111]. However, as noted by the health care providers in our study, it is essential that providers do not uncritically apply population-level health inequities data to clinical care among individual racially minoritized patients, which could lead to stereotyping, making erroneous assumptions, and screening patients from racially minoritized groups more frequently than necessary. Instead, providers should recognize the unique lived experiences of racially minoritized individuals and the structural, social, and economic factors that shape them [112, 113] by using person-centered, structurally-competent, and trauma-informed practices that acknowledge and address the impact of racism and emphasize shared decision-making, bodily autonomy, and agency [114–116]. This racism-conscious approach is not to be confused with race-based medicine, which is an essentialist and racist approach to health care that treats race as a biological variable rather than as a social and political construct.

To achieve this goal, health care provider education must meaningfully address the history of racism in medicine, including gynecology; provide community-informed guidance for addressing racism’s ongoing and contemporary effects in health care practice; and include training in person-centered care, structural competency, and trauma-informed care. For example, provider education should clearly emphasize the social and political nature of race and its link to racism and challenge inaccurate conceptions of race as an innate biological or behavioral factor [113, 117]; highlight the historical, structural, social, and economic causes of racialized health inequities rather than merely providing decontextualized statistics [118]; implement shared language, values, norms, practices, and behaviors that explicitly and meaningfully address racism in clinical care in a manner that is aligned with community experiences, contexts, priorities, and preferences [119]; and incorporate intentional and sustained efforts to encourage action and accountability on racism in health care settings [120]. Further, health care organizations should increase the proportion of health care providers from racially minoritized backgrounds [121] and establish institutional practices, policies, and norms that foster the safety, respect, and well-being of providers of color [119]. Finally, in order for these efforts to take root and be sustainable in the long-term, racism in institutional, local, state, and federal policies and institutional and societal practices and norms must also be addressed [13, 111, 122].

## Conclusions

In conclusion, there is a critical need to explicitly acknowledge and address racism in the development and implementation of cervical cancer screening clinical guidelines [8, 54–57, 70]. Indeed, most providers in our study described adopting a universal, one-size-fits-all approach based on research with predominately white individuals that did not consider the unique and specific lived experiences or contexts of racially minoritized individuals in relation to racism. Despite frequently acknowledging the existence of racialized cervical cancer inequities, few providers recognized the role of social inequities in shaping them, and none discussed the impact of racism. In contrast, key informants overwhelmingly recommended that providers adopt an approach to cervical cancer screening and follow-up care that recognizes the role of racism in shaping racialized cervical cancer and related social inequities, is developed in partnership with racially minoritized communities, and involves person-centered, structurally-competent, and trauma-informed practices that address racially minoritized peoples' unique lived experiences in historical and social context [8, 12, 54–57, 70, 116]. This racism-conscious approach is not to be confused with race-based medicine, which is an essentialist and racist approach to health care that treats race as a biological variable rather than as a social and political construct. These critical recommendations, along with addressing racism in social systems, institutions, practices, and policies more broadly [8, 111, 122], would help promote racial equity in cervical and other cancer outcomes in the U.S.

## Data Availability

The data used and analyzed in the present study are not available for use by other researchers per our informed consent form, which restricts the use of these data to this study. However, the in-depth interview guides used in this study are available from the corresponding author on reasonable request.
