# Correction: Barriers to and opportunities for advancing racial equity in cervical cancer screening in the United States

**DOI:** 10.1186/s12905-024-03276-9

**Published:** 2024-07-29

**Authors:** Madina Agénor, Madeline Noh, Rose Eiduson, Merrily LeBlanc, Emmett C. Line, Roberta E. Goldman, Jennifer Potter, S. Bryn Austin

**Affiliations:** 1 Department of Behavioral and Social Sciences, Brown University School of Public Health, Providence, RI USA; 2 Center for Health Promotion and Health Equity, Brown University School of Public Health, Providence, RI USA; 3 Department of Epidemiology, Brown University School of Public Health, MPH Box G-S121-4, Providence, RI 02912 USA; 4 The Fenway Institute, Fenway Health, Boston, MA USA; 5 Larner College of Medicine, University of Vermont, Burlington, VT USA; 6 Department of Sociology, Northeastern University, Boston, MA USA; 7 Teachers College, Columbia University, New York, NY USA; 8 Department of Family Medicine, Warren Alpert Medical School, Brown University, Providence, RI USA; 9 Department of Medicine, Harvard Medical School, Boston, MA USA; 10 Department of Medicine, Beth Israel Deaconess Medical Center, Boston, MA USA; 11 Department of Social and Behavioral Sciences, Harvard T.H. Chan School of Public Health, Boston, MA USA; 12 Division of Adolescent and Young Adult Medicine, Boston Children’s Hospital, Boston, MA USA; 13 Department of Pediatrics, Harvard Medical School, Boston, MA USA

**Correction: BMC Women’s Health (2024) 24:362**

10.1186/s12905-024-03151-7.

Following publication of the original article [1], the subheading was incorrectly given as ‘Theme 1. Adopting approach to cervical cancer screening’ but should have been ‘THEME 1. Adopting a one-size-fits-all approach to cervical cancer screening’.

The original article has been corrected.
